# Adipic acid–2,4-diamino-6-(4-meth­oxy­phen­yl)-1,3,5-triazine (1/2)

**DOI:** 10.1107/S1600536812038743

**Published:** 2012-09-15

**Authors:** Kaliyaperumal Thanigaimani, Ibrahim Abdul Razak, Suhana Arshad, Rathinavel Jagatheesan, Kulandaisamy Joseph Santhanaraj

**Affiliations:** a School of Physics, Universiti Sains Malaysia, 11800 USM, Penang, Malaysia; b Department of Chemistry, Angalamman College of Engineering and Technology, Siruganur, Tiruchirappalli 621 105, Tamil Nadu, India; c Department of Chemistry, St. Joseph’s College, Tiruchirappalli 620 002, Tamil Nadu, India

## Abstract

The asymmetric unit of the title compound, 2C_10_H_11_N_5_O·C_6_H_10_O_4_, consists of a 2,4-diamino-6-(4-meth­oxy­phen­yl)-1,3,5-triazine mol­ecule and one-half mol­ecule of adipic acid which lies about an inversion center. The triazine ring makes a dihedral angle of 12.89 (4)° with the adjacent benzene ring. In the crystal, the components are linked by N—H⋯O and O—H⋯N hydrogen bonds, thus generating a centrosymmetric 2 + 1 unit of triazine and adipic acid mol­ecules with *R*_2_^2^(8) motifs. The triazine mol­ecules are connected to each other by N—H⋯N hydrogen bonds, forming an *R*_2_^2^(8) motif and a supra­molecular ribbon along the *c* axis. The 2 + 1 units and the supra­molecular ribbons are further inter­linked by weak N—H⋯O, C—H⋯O and C—H⋯π inter­actions, resulting in a three-dimensional network.

## Related literature

For the biological activity of triazine derivatives, see: Bork *et al.* (2003 ▶). For bond-length data, see: Allen *et al.* (1987) ▶. For hydrogen-bond motifs, see: Bernstein *et al.* (1995 ▶). For the stability of the temperature controller used in the data collection, see: Cosier & Glazer (1986 ▶).
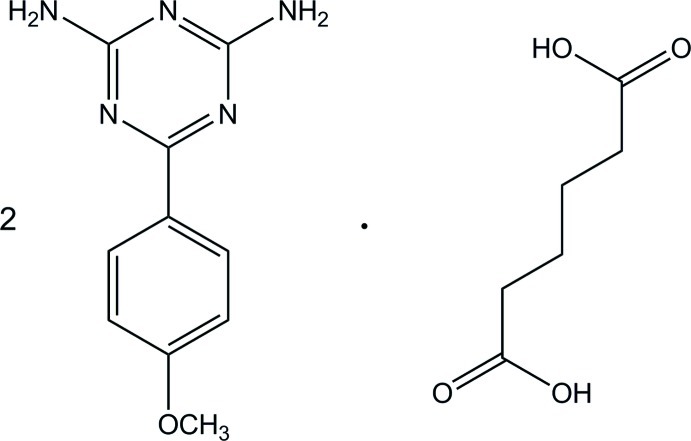


## Experimental

### 

#### Crystal data

2C_10_H_11_N_5_O·C_6_H_10_O_4_
*M* *_r_* = 580.62
Monoclinic, 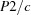

*a* = 15.9952 (9) Å
*b* = 7.3104 (5) Å
*c* = 12.0351 (7) Å
β = 96.912 (1)°
*V* = 1397.05 (15) Å^3^
*Z* = 2
Mo *K*α radiation
μ = 0.10 mm^−1^
*T* = 100 K
0.64 × 0.40 × 0.22 mm

#### Data collection

Bruker SMART APEXII DUO CCD area-detector diffractometer
Absorption correction: multi-scan (*SADABS*; Bruker, 2009 ▶) *T*_min_ = 0.938, *T*_max_ = 0.979
13850 measured reflections
4993 independent reflections
4263 reflections with *I* > 2σ(*I*)
*R*_int_ = 0.019

#### Refinement

*R*[*F* ^2^ > 2σ(*F* ^2^)] = 0.039
*wR*(*F* ^2^) = 0.126
*S* = 1.08
4993 reflections
211 parameters
H atoms treated by a mixture of independent and constrained refinement
Δρ_max_ = 0.49 e Å^−3^
Δρ_min_ = −0.22 e Å^−3^

### 

Data collection: *APEX2* (Bruker, 2009 ▶); cell refinement: *SAINT* (Bruker, 2009 ▶); data reduction: *SAINT*; program(s) used to solve structure: *SHELXTL* (Sheldrick, 2008 ▶); program(s) used to refine structure: *SHELXTL*; molecular graphics: *SHELXTL*; software used to prepare material for publication: *SHELXTL* and *PLATON* (Spek, 2009 ▶).

## Supplementary Material

Crystal structure: contains datablock(s) global, I. DOI: 10.1107/S1600536812038743/is5192sup1.cif

Structure factors: contains datablock(s) I. DOI: 10.1107/S1600536812038743/is5192Isup2.hkl

Supplementary material file. DOI: 10.1107/S1600536812038743/is5192Isup3.cml

Additional supplementary materials:  crystallographic information; 3D view; checkCIF report

## Figures and Tables

**Table 1:** Hydrogen-bond geometry (Å, °) *Cg*1 is the centroid of the N1/C2/N3/C4/N5/C6 triazine ring.

*D*—H⋯*A*	*D*—H	H⋯*A*	*D*⋯*A*	*D*—H⋯*A*
N2—H1*N*2⋯N5^i^	0.894 (15)	2.051 (15)	2.9438 (10)	177.7 (14)
N2—H2*N*2⋯O2^i^	0.868 (16)	2.336 (16)	2.9891 (10)	132.2 (13)
N4—H1*N*4⋯O2^ii^	0.901 (15)	2.021 (15)	2.9142 (11)	170.9 (13)
N4—H2*N*4⋯N1^iii^	0.906 (16)	2.245 (16)	3.1456 (10)	172.5 (14)
O1—H1*O*1⋯N3^iv^	0.953 (15)	1.728 (15)	2.6655 (10)	167.5 (15)
C13—H13*A*⋯O3^v^	0.98	2.53	3.3997 (12)	148
C15—H15*A*⋯*Cg*1	0.99	2.83	3.598	135

Symmetry codes: (i) 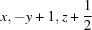; (ii) 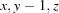; (iii) 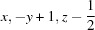; (iv) 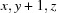; (v) 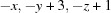.

**Fig. 1.**
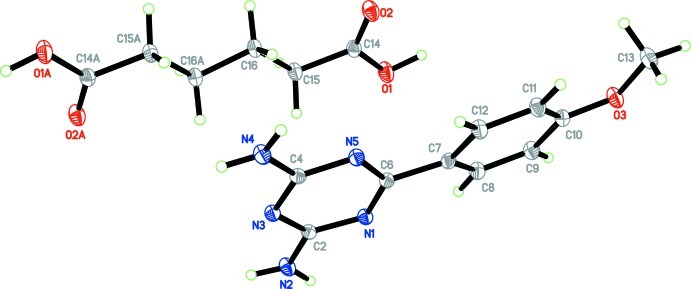
The molecular structure of the title compound, showing 50% probability displacement ellipsoids and the atom-numbering scheme.

**Fig. 2.**
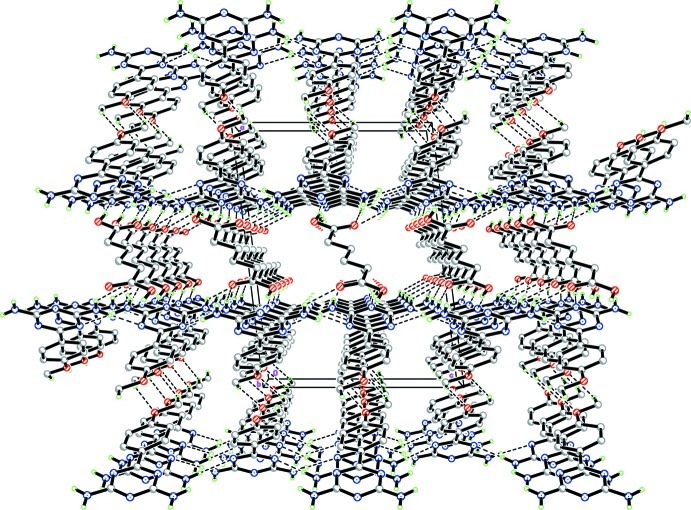
The crystal packing of the title compound. The H atoms not involved in the intermolecular interactions (dashed lines) have been omitted for clarity.

## supplementary crystallographic information

### Comment

Triazine derivatives show antitumour activity as well as a broad range of biological activities, such as anti-angiogenesis and antimicrobial effects (Bork *et al.*, 2003). Adipic acid is used to ester for platicizers and as food additive. In order to study some interesting hydrogen bonding interaction, the synthesis and structure of the title compound, (I), is presented here.

The asymmetric unit of the title compound, (I), consists of a 2,4-diamino-6-(4-methoxyphenyl)-1,3,5-triazine molecule and a half of the adipic acid molecule (Fig. 1). The planar adipic acid molecule is centrosymmetric with the mid-point of the central C—C bond located at an inversion center. The C14—O2 bond distance of 1.2248 (10) Å is much shorter than the C14—O1 bond distance of 1.3199 (11) Å, indicating that the carboxyl group is not deprotonated in the crystal structure. The dihedral angle between the triazine ring [N1/C2/N3/C4/N5/C6, maximum deviation = 0.010 (1) Å for atoms N4 & C4] and the plane formed by the adipic acid molecule (O1/O2/C14-C16) is 2.14 (4)°. The triazine ring forms dihedral angle of 12.89 (4)° with the benzene ring (C7–C12). The conformation of adipic acid can be described by the two torsion angles C14—C15—C16—C16A of -175.20 (7)° and C15—C16—C16A—C15A of 179.98 (9)°. The bond lengths (Allen *et al.*, 1987) and angles are normal.

In the crystal (Fig. 2). the triazine molecules are base-paired [with a graph set (Bernstein *et al.*, 1995) of R_2_^2^(8)] on either side *via* N2—H1N2···N5^i^ and N4—H2N4···N1^iii^ hydrogen bonds (symmetry codes in Table 1), forming a supramolecular ribbon. Each triazine molecule interacts with the carboxyl group of adipic acid molecule *via* N4—H1N4···O2^ii^ and O1—H1O1···N3^iV^ hydrogen bonds (symmetry codes in Table 1), generating R_2_^2^(8) motifs (Bernstein *et al.*, 1995). The supramolecular ribbons are linked by N2—H2N2···O2^i^ hydrogen bonds, resulting a three-dimensional network. The crystal structure are further stabilized by weak C13—H13A···O3^v^ and C—H···π interactions (Table 1) involving the N1/C2/N3/C4/N5/C6 (centroid *Cg*1) ring.

### Experimental

A hot methanol solution (20 ml) of 2,4-diamino-6-(4-methoxyphenyl)-1,3,5-triazine (54 mg Aldrich) and adipic acid (36 mg Loba) was warmed for a half an hour over a water bath. The resulting solution was allowed to cool slowly at room temperature. After a few days colourless block crystals were obtained.

### Refinement

Atoms H1N2, H2N2, H1N4, H2N4 and H1O1 were located from a difference Fourier maps and refined freely [N—H = 0.894 (15), 0.868 (16), 0.903 (15) and 0.906 (16) Å and O—H = 0.952 (17) Å]. The remaining H atoms were positioned geometrically (C—H= 0.95–0.99 Å) and were refined using a riding model, with *U*_iso_(H) = 1.2*U*_eq_(C) or 1.5*U*_eq_(methyl C). A rotating-group model was used for the methyl group.

### Crystal data

**Table:**

2C_10_H_11_N_5_O·C_6_H_10_O_4_	*F*(000) = 612
*M**_r_* = 580.62	*D*_x_ = 1.380 Mg m^−^^3^
Monoclinic, *P*2/*c*	Mo *K*α radiation, λ = 0.71073 Å
Hall symbol: -P 2yc	Cell parameters from 7141 reflections
*a* = 15.9952 (9) Å	θ = 2.8–32.4°
*b* = 7.3104 (5) Å	µ = 0.10 mm^−^^1^
*c* = 12.0351 (7) Å	*T* = 100 K
β = 96.912 (1)°	Block, colourless
*V* = 1397.05 (15) Å^3^	0.64 × 0.40 × 0.22 mm
*Z* = 2	

### Data collection

**Table:**

Bruker SMART APEXII DUO CCD area-detector diffractometer	4993 independent reflections
Radiation source: fine-focus sealed tube	4263 reflections with *I* > 2σ(*I*)
Graphite monochromator	*R*_int_ = 0.019
φ and ω scans	θ_max_ = 32.6°, θ_min_ = 2.8°
Absorption correction: multi-scan (*SADABS*; Bruker, 2009)	*h* = −24→24
*T*_min_ = 0.938, *T*_max_ = 0.979	*k* = −10→11
13850 measured reflections	*l* = −18→17

### Refinement

**Table:**

Refinement on *F*^2^	Primary atom site location: structure-invariant direct methods
Least-squares matrix: full	Secondary atom site location: difference Fourier map
*R*[*F*^2^ > 2σ(*F*^2^)] = 0.039	Hydrogen site location: inferred from neighbouring sites
*wR*(*F*^2^) = 0.126	H atoms treated by a mixture of independent and constrained refinement
*S* = 1.08	*w* = 1/[σ^2^(*F*_o_^2^) + (0.0737*P*)^2^ + 0.2639*P*] where *P* = (*F*_o_^2^ + 2*F*_c_^2^)/3
4993 reflections	(Δ/σ)_max_ = 0.001
211 parameters	Δρ_max_ = 0.49 e Å^−^^3^
0 restraints	Δρ_min_ = −0.22 e Å^−^^3^

### Special details

**Table:**

Experimental. The crystal was placed in the cold stream of an Oxford Cryosystems Cobra open-flow nitrogen cryostat (Cosier & Glazer, 1986) operating at 100.0 (1) K.
Geometry. All e.s.d.'s (except the e.s.d. in the dihedral angle between two l.s. planes) are estimated using the full covariance matrix. The cell e.s.d.'s are taken into account individually in the estimation of e.s.d.'s in distances, angles and torsion angles; correlations between e.s.d.'s in cell parameters are only used when they are defined by crystal symmetry. An approximate (isotropic) treatment of cell e.s.d.'s is used for estimating e.s.d.'s involving l.s. planes.
Refinement. Refinement of *F*^2^ against ALL reflections. The weighted *R*-factor *wR* and goodness of fit *S* are based on *F*^2^, conventional *R*-factors *R* are based on *F*, with *F* set to zero for negative *F*^2^. The threshold expression of *F*^2^ > σ(*F*^2^) is used only for calculating *R*-factors(gt) *etc*. and is not relevant to the choice of reflections for refinement. *R*-factors based on *F*^2^ are statistically about twice as large as those based on *F*, and *R*- factors based on ALL data will be even larger.

### Fractional atomic coordinates and isotropic or equivalent isotropic displacement parameters (Å^2^)

**Table:**

	*x*	*y*	*z*	*U*_iso_*/*U*_eq_	
O1	0.38325 (5)	0.96141 (10)	0.63437 (6)	0.02222 (15)	
O2	0.39639 (4)	0.93287 (9)	0.45203 (5)	0.01924 (14)	
O3	0.06629 (4)	1.28973 (9)	0.50394 (6)	0.01951 (14)	
N1	0.24094 (4)	0.53425 (10)	0.66667 (5)	0.01295 (13)	
N2	0.30198 (5)	0.29976 (11)	0.77505 (6)	0.01611 (14)	
N3	0.30677 (4)	0.28034 (10)	0.58551 (5)	0.01296 (13)	
N4	0.30393 (5)	0.26981 (11)	0.39410 (6)	0.01582 (14)	
N5	0.24267 (4)	0.51829 (10)	0.46927 (5)	0.01320 (13)	
C2	0.28291 (5)	0.37227 (11)	0.67348 (6)	0.01221 (14)	
C4	0.28388 (5)	0.35704 (11)	0.48465 (6)	0.01227 (14)	
C6	0.22262 (4)	0.59896 (11)	0.56262 (6)	0.01197 (14)	
C7	0.17790 (5)	0.77580 (12)	0.54733 (6)	0.01294 (15)	
C8	0.16892 (5)	0.89239 (12)	0.63761 (7)	0.01547 (15)	
H8A	0.1896	0.8550	0.7114	0.019*	
C9	0.13032 (5)	1.06136 (12)	0.62062 (7)	0.01631 (16)	
H9A	0.1246	1.1387	0.6827	0.020*	
C10	0.09974 (5)	1.11861 (12)	0.51240 (7)	0.01452 (15)	
C11	0.10537 (5)	1.00202 (13)	0.42193 (7)	0.01636 (16)	
H11A	0.0828	1.0379	0.3485	0.020*	
C12	0.14445 (5)	0.83235 (12)	0.44012 (7)	0.01592 (15)	
H12A	0.1485	0.7534	0.3783	0.019*	
C13	0.04003 (6)	1.35766 (14)	0.39371 (8)	0.02142 (18)	
H13A	0.0194	1.4833	0.3986	0.032*	
H13B	−0.0052	1.2802	0.3572	0.032*	
H13C	0.0878	1.3560	0.3499	0.032*	
C14	0.40924 (5)	0.87581 (12)	0.54826 (7)	0.01502 (15)	
C15	0.45564 (5)	0.70071 (12)	0.58066 (7)	0.01631 (16)	
H15A	0.4198	0.6232	0.6230	0.020*	
H15B	0.5074	0.7305	0.6311	0.020*	
C16	0.47996 (5)	0.59149 (12)	0.48172 (7)	0.01603 (15)	
H16A	0.5201	0.6637	0.4430	0.019*	
H16B	0.4291	0.5694	0.4279	0.019*	
H1N2	0.2826 (9)	0.353 (2)	0.8337 (12)	0.030 (4)*	
H2N2	0.3333 (10)	0.203 (2)	0.7873 (13)	0.036 (4)*	
H1N4	0.3319 (9)	0.163 (2)	0.4039 (12)	0.027 (3)*	
H2N4	0.2840 (9)	0.316 (2)	0.3262 (13)	0.035 (4)*	
H1O1	0.3554 (11)	1.070 (2)	0.6062 (14)	0.041 (4)*	

### Atomic displacement parameters (Å^2^)

**Table:**

	*U*^11^	*U*^22^	*U*^33^	*U*^12^	*U*^13^	*U*^23^
O1	0.0314 (3)	0.0187 (3)	0.0169 (3)	0.0107 (3)	0.0047 (2)	0.0004 (2)
O2	0.0258 (3)	0.0157 (3)	0.0157 (3)	0.0051 (2)	0.0006 (2)	−0.0016 (2)
O3	0.0257 (3)	0.0149 (3)	0.0178 (3)	0.0063 (2)	0.0016 (2)	0.0010 (2)
N1	0.0156 (3)	0.0133 (3)	0.0100 (3)	0.0017 (2)	0.0017 (2)	0.0006 (2)
N2	0.0233 (3)	0.0151 (3)	0.0097 (3)	0.0037 (3)	0.0011 (2)	0.0014 (2)
N3	0.0158 (3)	0.0130 (3)	0.0102 (3)	0.0015 (2)	0.0019 (2)	0.0001 (2)
N4	0.0223 (3)	0.0147 (3)	0.0106 (3)	0.0037 (3)	0.0026 (2)	−0.0006 (2)
N5	0.0155 (3)	0.0137 (3)	0.0105 (3)	0.0024 (2)	0.0022 (2)	−0.0002 (2)
C2	0.0131 (3)	0.0128 (3)	0.0106 (3)	−0.0002 (2)	0.0008 (2)	0.0005 (2)
C4	0.0130 (3)	0.0128 (3)	0.0111 (3)	−0.0005 (2)	0.0016 (2)	−0.0003 (2)
C6	0.0122 (3)	0.0135 (3)	0.0102 (3)	0.0006 (2)	0.0014 (2)	0.0001 (2)
C7	0.0132 (3)	0.0143 (4)	0.0115 (3)	0.0019 (3)	0.0023 (2)	0.0002 (3)
C8	0.0170 (3)	0.0172 (4)	0.0121 (3)	0.0024 (3)	0.0010 (2)	−0.0003 (3)
C9	0.0193 (3)	0.0158 (4)	0.0139 (3)	0.0025 (3)	0.0021 (3)	−0.0019 (3)
C10	0.0142 (3)	0.0138 (4)	0.0156 (3)	0.0016 (3)	0.0020 (2)	0.0005 (3)
C11	0.0187 (3)	0.0169 (4)	0.0132 (3)	0.0038 (3)	0.0010 (3)	0.0010 (3)
C12	0.0190 (3)	0.0168 (4)	0.0119 (3)	0.0040 (3)	0.0016 (2)	−0.0002 (3)
C13	0.0247 (4)	0.0187 (4)	0.0202 (4)	0.0055 (3)	0.0001 (3)	0.0037 (3)
C14	0.0149 (3)	0.0127 (4)	0.0173 (3)	0.0007 (3)	0.0012 (2)	−0.0017 (3)
C15	0.0177 (3)	0.0132 (4)	0.0179 (3)	0.0033 (3)	0.0017 (3)	0.0004 (3)
C16	0.0164 (3)	0.0130 (4)	0.0185 (3)	0.0032 (3)	0.0011 (3)	−0.0003 (3)

### Geometric parameters (Å, º)

**Table:**

O1—C14	1.3199 (10)	C7—C8	1.4018 (11)
O1—H1O1	0.952 (17)	C8—C9	1.3851 (12)
O2—C14	1.2249 (10)	C8—H8A	0.9500
O3—C10	1.3595 (10)	C9—C10	1.3991 (11)
O3—C13	1.4314 (11)	C9—H9A	0.9500
N1—C6	1.3377 (10)	C10—C11	1.3942 (12)
N1—C2	1.3587 (10)	C11—C12	1.3944 (12)
N2—C2	1.3337 (10)	C11—H11A	0.9500
N2—H1N2	0.894 (15)	C12—H12A	0.9500
N2—H2N2	0.868 (16)	C13—H13A	0.9800
N3—C4	1.3472 (10)	C13—H13B	0.9800
N3—C2	1.3473 (10)	C13—H13C	0.9800
N4—C4	1.3347 (10)	C14—C15	1.5072 (12)
N4—H1N4	0.903 (15)	C15—C16	1.5226 (12)
N4—H2N4	0.906 (16)	C15—H15A	0.9900
N5—C6	1.3415 (10)	C15—H15B	0.9900
N5—C4	1.3524 (11)	C16—C16^i^	1.5251 (17)
C6—C7	1.4783 (11)	C16—H16A	0.9900
C7—C12	1.3985 (11)	C16—H16B	0.9900
			
C14—O1—H1O1	107.1 (10)	O3—C10—C9	115.80 (7)
C10—O3—C13	117.23 (7)	C11—C10—C9	119.73 (8)
C6—N1—C2	114.56 (7)	C10—C11—C12	119.41 (7)
C2—N2—H1N2	119.2 (10)	C10—C11—H11A	120.3
C2—N2—H2N2	122.8 (10)	C12—C11—H11A	120.3
H1N2—N2—H2N2	118.0 (14)	C11—C12—C7	121.49 (7)
C4—N3—C2	115.37 (7)	C11—C12—H12A	119.3
C4—N4—H1N4	118.1 (9)	C7—C12—H12A	119.3
C4—N4—H2N4	117.7 (10)	O3—C13—H13A	109.5
H1N4—N4—H2N4	123.8 (14)	O3—C13—H13B	109.5
C6—N5—C4	115.48 (7)	H13A—C13—H13B	109.5
N2—C2—N3	117.83 (7)	O3—C13—H13C	109.5
N2—C2—N1	117.29 (7)	H13A—C13—H13C	109.5
N3—C2—N1	124.88 (7)	H13B—C13—H13C	109.5
N4—C4—N3	118.09 (7)	O2—C14—O1	123.24 (8)
N4—C4—N5	117.76 (7)	O2—C14—C15	123.73 (7)
N3—C4—N5	124.15 (7)	O1—C14—C15	113.04 (7)
N1—C6—N5	125.52 (7)	C14—C15—C16	114.03 (7)
N1—C6—C7	118.30 (7)	C14—C15—H15A	108.7
N5—C6—C7	116.17 (7)	C16—C15—H15A	108.7
C12—C7—C8	118.16 (8)	C14—C15—H15B	108.7
C12—C7—C6	119.94 (7)	C16—C15—H15B	108.7
C8—C7—C6	121.89 (7)	H15A—C15—H15B	107.6
C9—C8—C7	120.88 (7)	C15—C16—C16^i^	111.86 (9)
C9—C8—H8A	119.6	C15—C16—H16A	109.2
C7—C8—H8A	119.6	C16^i^—C16—H16A	109.2
C8—C9—C10	120.26 (7)	C15—C16—H16B	109.2
C8—C9—H9A	119.9	C16^i^—C16—H16B	109.2
C10—C9—H9A	119.9	H16A—C16—H16B	107.9
O3—C10—C11	124.46 (7)		
			
C4—N3—C2—N2	178.22 (7)	C12—C7—C8—C9	−1.99 (12)
C4—N3—C2—N1	−1.99 (11)	C6—C7—C8—C9	176.85 (7)
C6—N1—C2—N2	−179.18 (7)	C7—C8—C9—C10	−0.16 (12)
C6—N1—C2—N3	1.04 (11)	C13—O3—C10—C11	−4.03 (12)
C2—N3—C4—N4	−178.37 (7)	C13—O3—C10—C9	175.58 (7)
C2—N3—C4—N5	2.48 (11)	C8—C9—C10—O3	−177.20 (7)
C6—N5—C4—N4	178.87 (7)	C8—C9—C10—C11	2.43 (12)
C6—N5—C4—N3	−1.97 (11)	O3—C10—C11—C12	177.11 (8)
C2—N1—C6—N5	−0.45 (12)	C9—C10—C11—C12	−2.49 (12)
C2—N1—C6—C7	−179.26 (7)	C10—C11—C12—C7	0.30 (12)
C4—N5—C6—N1	0.90 (12)	C8—C7—C12—C11	1.92 (12)
C4—N5—C6—C7	179.73 (7)	C6—C7—C12—C11	−176.95 (7)
N1—C6—C7—C12	−168.63 (7)	O2—C14—C15—C16	−4.38 (12)
N5—C6—C7—C12	12.46 (11)	O1—C14—C15—C16	175.24 (7)
N1—C6—C7—C8	12.56 (11)	C14—C15—C16—C16^i^	−175.20 (8)
N5—C6—C7—C8	−166.36 (7)		

Symmetry code: (i) −*x*+1, −*y*+1, −*z*+1.

### Hydrogen-bond geometry (Å, º)

*Cg*1 is the centroid of the N1/C2/N3/C4/N5/C6 triazine ring.

**Table:**

*D*—H···*A*	*D*—H	H···*A*	*D*···*A*	*D*—H···*A*
N2—H1*N*2···N5^ii^	0.894 (15)	2.051 (15)	2.9438 (10)	177.7 (14)
N2—H2*N*2···O2^ii^	0.868 (16)	2.336 (16)	2.9891 (10)	132.2 (13)
N4—H1*N*4···O2^iii^	0.901 (15)	2.021 (15)	2.9142 (11)	170.9 (13)
N4—H2*N*4···N1^iv^	0.906 (16)	2.245 (16)	3.1456 (10)	172.5 (14)
O1—H1*O*1···N3^v^	0.953 (15)	1.728 (15)	2.6655 (10)	167.5 (15)
C13—H13*A*···O3^vi^	0.98	2.53	3.3997 (12)	148
C15—H15*A*···*Cg*1	0.99	2.83	3.598	135

Symmetry codes: (ii) *x*, −*y*+1, *z*+1/2; (iii) *x*, *y*−1, *z*; (iv) *x*, −*y*+1, *z*−1/2; (v) *x*, *y*+1, *z*; (vi) −*x*, −*y*+3, −*z*+1.
